# Thermal Stimulation in Addition to Conventional Therapy on Functional Recovery After Stroke: A Case Series

**DOI:** 10.3390/healthcare14213673

**Published:** 2026-10-29

**Authors:** Daniela Celi-Lalama, Felipe Tohme-Sussmann, Felipe Araya-Quintanilla, Iván Yánez-Ortiz, Wilmer Esparza-Yánez, Juan Pablo Hervás-Pérez, Héctor Gutiérrez-Espinoza

**Affiliations:** 1 Escuela Internacional de Doctorado, Universidad Camilo José Cela, 28692 Madrid, Spain; 2 Faculty of Medical, Health and Life Sciences, International University of Ecuador, UIDE, Quito 170113, Ecuador; ivyanezor@uide.edu.ec; 3 Servicio de Neurología, Hospital de Especialidades Eugenio Espejo, Quito 170403, Ecuador; feliptohme@gmail.com; 4 Escuela de Kinesiología, Facultad de Ciencias de la Rehabilitación y Calidad de Vida, Universidad San Sebastián, Santiago 7510157, Chile; felipe.arayaq@uss.cl; 5 Unité de Médecine Gériatrique Aiguë (UMGA), CHU de Besançon, F-25000 Besançon, France; wesparzayanez@chu-besancon.fr; 6 Analytical Chemistry Unit, Department of Chemistry in Pharmaceutical Sciences, Faculty of Pharmacy, Complutense University of Madrid, 28040 Madrid, Spain; jphervas@ucm.es; 7 Faculty of Education, Universidad Autónoma de Chile, Santiago 7500912, Chile; hector.gutierrez@uautonoma.cl; 8 Universidad Espíritu Santo, Samborondón 090152, Ecuador

**Keywords:** stroke, thermal stimulation, conventional therapy, functional recovery, quality of life

## Abstract

Background/Objectives: To describe changes in motor function (FMA), functional independence (BI), and quality of life (SS-QOL) associated with thermal stimulation in addition to conventional therapy in first-ever stroke. Methods: A case series with 3-month follow-up was conducted in patients during the acute and subacute phases of first-ever stroke. Treatment consisted of thermal stimulation (alternating heat > 45 °C and cold < 15 °C applied to the hemiplegic hand and foot) followed by conventional therapy for 12 weeks (5 sessions/week). Outcomes were assessed at 3 times: baseline (T0), post-treatment (T1), and 3-month follow-up (T2) using the MAS, MRC, FMA, BI, and SS-QOL. Results: In total, 51 post-stroke patients were included (33 women, 18 men; mean age, 59.3 ± 18.5 years). Muscle strength improved significantly across all four evaluated muscle groups from T0 to T1, with gains maintained at T2 (*p* < 0.0001), while muscle tone showed no statistically significant changes. FMA upper extremity scores increased by 34.88 points (95% CI: 31.85–37.91) and lower extremity scores by 11.00 points (95% CI: 9.56–12.44) at T1 (both *p* < 0.0001), with gains sustained at T2. The BI improved by 24.71 points (95% CI: 19.40–30.05; *p* < 0.0001) at T1, with no further significant change at T2. SS-QOL scores increased by 63.90 points (95% CI: 51.60–76.30; *p* < 0.0001) at T1 and continued to improve at T2 (+78.30 points; 95% CI: 65.90–90.60). All functional outcomes showed large effect sizes at T2 (η^2^ > 0.14). Conclusions: thermal stimulation added to conventional therapy was associated with statistically significant and sustained changes in motor function, functional independence, and quality of life after first-ever stroke, supporting its consideration as a complementary neurorehabilitation strategy.

## 1. Introduction

Stroke is a sudden-onset neurological disorder of vascular origin that is regarded as a medical emergency because of its high mortality rate and the significant disability it causes [1]. The consequences include motor and cognitive impairments, as well as deficits in language and swallowing, which have a substantial impact on the autonomy and quality of life of affected patients [2]. Globally, there has been a sustained increase in the incidence of stroke, with a 70% rise in incidence and an 85% increase in prevalence between 1990 and 2019. This positions stroke as the second and third leading cause of death and disability, respectively [3]. In Latin America and the Caribbean, the estimated rate is 32 cases per 1000 population, with an annual incidence of 255 per 100,000 inhabitants. In Ecuador, stroke has progressively emerged as a significant cause of mortality, ranking among the leading causes of death in recent decades, with 4577 deaths recorded in 2019 [4,5].

The clinical manifestations of stroke predominantly include motor deficits, which affect approximately 80% of patients [6]. These deficits include alterations in muscle tone that progress from flaccidity in the acute phase to hypertonia and spasticity in later stages, associated with reflex hyperexcitability [7]. Simultaneously, voluntary motor control is compromised, with loss of selectivity and the presence of abnormal synergies and muscle coactivation, resulting in weakness and hemiparesis on the affected side of the body [8]. Furthermore, up to 40% of patients experience moderate disability and up to 30–40% experience severe disability [9].

The treatment of stroke requires a prompt, multidisciplinary approach that, in hemodynamically stable patients, can be initiated within the first 48 h following the event [10]. Conventional stroke therapy involves the integration of rehabilitation methods such as Bobath therapy, proprioceptive neuromuscular facilitation, and motor relearning, complemented by techniques aimed at optimizing motor and sensory functions [11]. In this context, thermal stimulation has emerged as a viable therapeutic modality that can be incorporated into rehabilitation protocols, given its capacity to modulate neuromuscular excitability, facilitate motor responses, and promote neural reorganization. This makes it a useful addition to conventional therapy strategies [12,13].

Alternating thermal stimulation involves applying temperatures exceeding 45 °C (heat) and below 15 °C (cold), which engage both innocuous and noxious thermal receptors [14]. Evidence shows that thermal stimulation activates various sensory and motor regions, including the somatosensory cortex, supplementary motor area, anterior cingulate cortex, and thalamus [15,16]. Thermal stimulation not only elicits sensory responses but also induces reflex motor activity [14]. This may enhance the function of the affected limbs and support motor recovery following stroke, in a manner analogous to the benefits conferred by aerobic exercise [13,17]. Thermal stimulation serves as an adjunct to conventional therapy, aiming to enhance corticomotor excitability and promote brain reorganization, thereby increasing motor map size [14,15]. Additionally, a systematic review and meta-analysis revealed that adding thermal stimulation to conventional therapy significantly improves motor function, especially in the lower limbs and gait [12].

The variability in the frequency and dosage of thermal stimulation reported across studies introduces heterogeneity that may limit the consistency and generalizability of findings, highlighting the need for controlled interventions [12,14]. In this context, a case series involving patients in the subacute phase following a first stroke can provide a preliminary characterization of the functional outcomes associated with the treatment. Such a study can offer descriptive information that, although it does not allow causal relationships to be established or the influence of spontaneous recovery to be excluded, helps define the clinical response profile of this intervention. Three months of follow-up is particularly important because it facilitates assessment of the sustainability of functional improvements and the detection of short-term changes in motor recovery. Accordingly, the present study aims to evaluate the effects of thermal stimulation in addition to conventional therapy on motor function, independence, and quality of life in patients with a first stroke.

## 2. Materials and Methods

### 2.1. Design/Setting

A case series was conducted in accordance with the guidelines of the Strengthening the Reporting of Observational Studies in Epidemiology (STROBE) statement [18]. This study received approval from the Ethics Committee of the University of the Americas in May 2024 (ID:2024-OBS-008). From August 2024 to August 2025, a total of 51 patients were consecutively and prospectively recruited using a non-probability sampling strategy. All patients provided informed consent, as approved by the ethics committee. The thermal stimulation protocol evaluated in this study had already been implemented by the center as part of its standard clinical care for first-ever stroke patients prior to this study, based on prior findings from the authors’ own meta-analysis; the investigators did not design or assign the intervention but documented the outcomes of patients who received this routine protocol during the study period.

### 2.2. Patients

**Figure 1:**
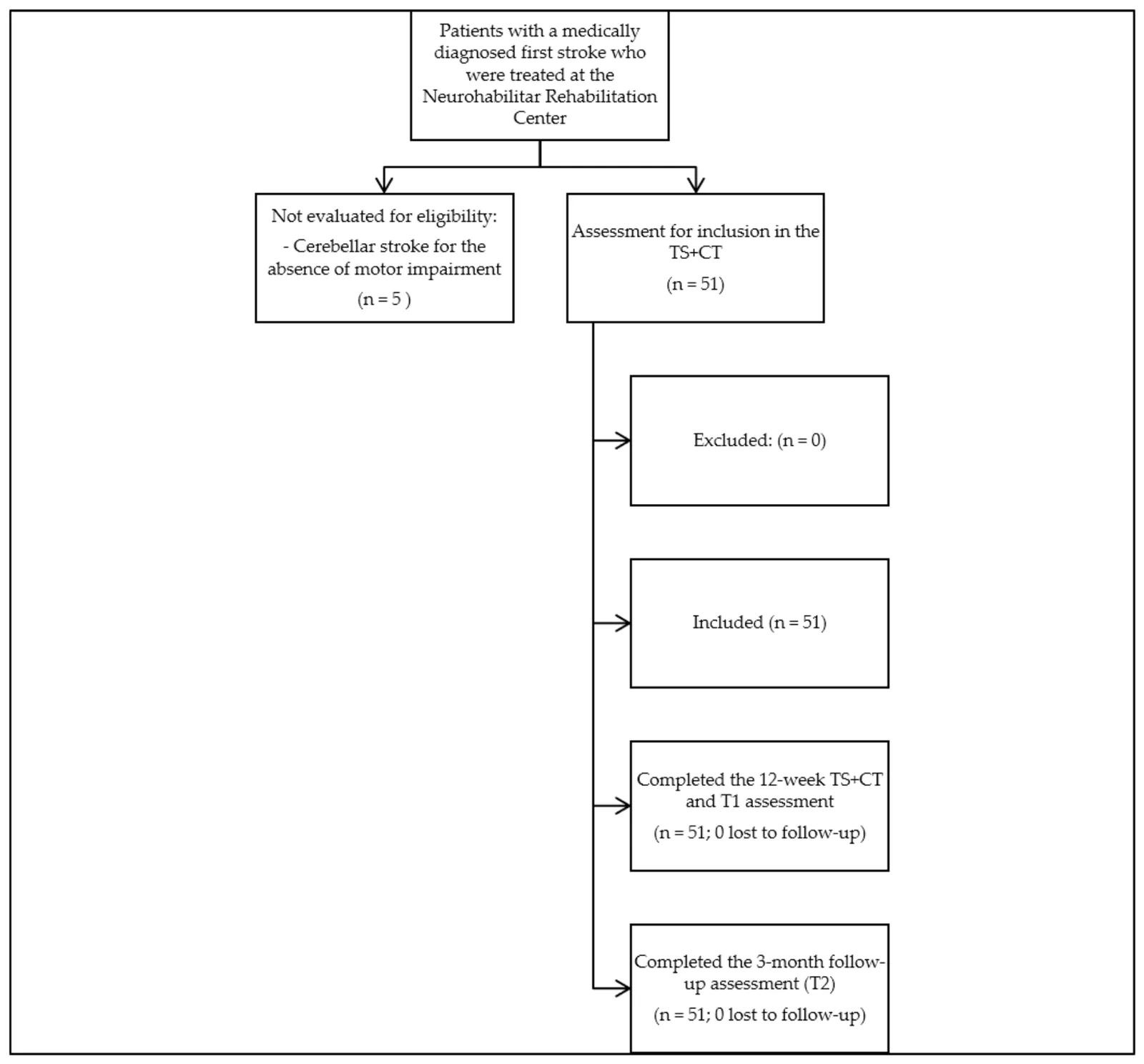
Participant flow diagram (STROBE), including the diagnosis routing of patients with cerebellar infarct who were not evaluated for this study.

Patients over 18 years of age presenting with hemiparesis as a motor impairment were recruited at the *Neurohabilitar* Rehabilitation Center, Quito, Ecuador. To be eligible for participation, patients had to meet the following conditions: a first ischemic or hemorrhagic stroke with a maximum duration of 3 months, diagnosed by a medical team in a hospital setting using a neurological deficit assessment algorithm that included clinical evaluation with the National Institutes of Health Stroke Scale and noncontrast computed tomography of the brain [19]. Patients with major cognitive deficits (Montreal Cognitive Assessment score < 20), communication impairments preventing them from following directions (e.g., deafness, aphasia), hemodynamic instability, skin conditions that prevented thermal stimulation, acute inflammation, deep vein thrombosis, or Raynaud’s disease were excluded. Patients with a diagnosis of cerebellar infarct were not evaluated for this study at all; based on this diagnosis alone, they were redirected at intake to other center protocols focused on different clinical characteristics, without undergoing any study-specific assessment. The participant flowchart is shown in Figure 1.

### 2.3. Interventions

The rehabilitation program consisted of thermal stimulation in addition to conventional therapy, with 60 min sessions conducted five times weekly over a period of 12 weeks.

#### 2.3.1. Thermal Stimulation

The thermal stimulation phase consisted of the alternating application of thermal stimuli to the affected side of the body, using heat (>45 °C) for 15 s, followed by a cold stimulus (<15 °C) and a 30 s rest period. Compresses were placed on the palm of the hand and the sole of the foot on the hemiparetic side. This cycle was repeated 10 consecutive times, with two complete cycles performed per session. To ensure thermal consistency, the compresses were replaced up to four times per cycle, and the temperature was verified using a digital thermometer to ensure that the established temperature ranges (>45 °C for heat and <15 °C for cold) were maintained throughout the intervention). Detailed protocol information is provided in the Supplementary Materials (Supplementary Table S1).

#### 2.3.2. Conventional Therapy

Conventional therapy emphasizes activities grounded in neurophysiological principles, using various techniques such as Bobath, proprioceptive neuromuscular facilitation, and motor relearning [13]. These techniques are coordinated with exercises performed in different positions—prone, supine, lateral, on all fours, kneeling, and standing—to facilitate postural control, activate muscle chains, modulate hypertonia, strengthen weak muscles, correct posture, promote symmetry, and enhance body awareness. Additionally, the approach aims to foster motor learning through automatisms and mobilization [20,21]. Details of these activities are provided in the Supplementary Materials (Supplementary Table S1).

The standardized physiotherapy program was administered by two physiotherapists, each with 15 years of clinical experience in neurological rehabilitation. Adherence and adverse events were monitored and recorded in each participant’s data collection notebook. No adverse events including burns or skin lesion were recorded throughout the intervention period.

### 2.4. Outcome Measures

Outcome assessment at three time points: at baseline (T0), after the 12-week treatment (T1), and at the 3-month follow-up (T2) were performed by two physiotherapists from the center, each with more than 15 years of clinical experience in neurorehabilitation, who were not involved in treatment delivery. As this was a single group design in which all participants received the same intervention protocol, there was no treatment allocation for patients or assessors to be blinded to.

#### 2.4.1. Sociodemographic Characteristics

Age, sex, and employment status before the stroke were evaluated. Comorbidity data were also assessed, including hypertension (defined by the World Health Organization/Pan American Health Organization as systolic blood pressure ≥ 140 mmHg and diastolic blood pressure ≥ 90 mmHg or the use of antihypertensive pharmacological treatment) [22]. Diabetes mellitus was defined as fasting blood glucose ≥ 126 mg/dL, hemoglobin A1c > 6.5%, or treatment with a hypoglycemic agent or insulin (American Diabetes Association) [23]. Dyslipidemia was defined as abnormal blood lipid levels, including total cholesterol > 200 mg/dL and triglycerides > 150 mg/dL [24]. Atrial fibrillation was defined as irregular heartbeats, absence of P waves, and fibrillation [25]. Coronary artery disease was described as a condition in which the arteries of the heart are blocked by fatty plaques [26]. Active smoking was defined as current cigarette smoking, and depression as a mood disorder causing persistent sadness and loss of interest in activities [27]. Comorbidity data were collected by patient self-report during the interview, as medical records were held by the hospitals where patients had been treated and were not accessible to the research team.

#### 2.4.2. Anthropometric Variables

All patients were measured without footwear using the same scale and stadiometer to determine height (cm), weight (kg), and body mass index (BMI, kg/m^2^). BMI values were subsequently used to evaluate nutritional status in accordance with the standards established by the World Health Organization [28].

#### 2.4.3. Clinical Variables

Spasticity was assessed using the Modified Ashworth Scale (MAS) in muscle groups on the affected side of the body. In the upper limb, the elbow flexors (biceps brachii) were evaluated, while in the lower limb, the plantar flexors (gastrocnemius) were assessed. The MAS classifies muscle tone into six ordinal categories (0, 1, 1+, 2, 3, and 4), with higher scores indicating greater muscle tone [29]. The MAS is used for the clinical assessment of muscle tone in patients with stroke, with evidence supporting moderate to high inter- and intrarater reliability [30]. Muscle strength was assessed in the supine position using the Medical Research Council scale, which grades muscle strength on an ordinal scale from 0 to 5 based on the ability to move against gravity and resistance and has demonstrated adequate validity and reliability for the clinical assessment of muscle strength [31,32]. This assessment targeted four muscle groups: elbow flexors and wrist extensors in the upper limb and hip flexors and dorsiflexors in the lower limb [32].

#### 2.4.4. Functional Variables

##### Fugl-Meyer Assessment (FMA)

The domains of this scale pertaining to motor function of the upper and lower extremities were assessed. Each item is scored from 0 to 2 points: 2 indicates performance of the entire movement, 1 indicates partial performance of the movement, and 0 indicates inability to perform the movement. The maximum score for upper extremity motor function is 66 points, while that for lower extremity motor function is 34 points, for a total maximum score of 100 points. This instrument has demonstrated validity and reliability in the assessment of motor function in the upper and lower extremities [33].

##### Barthel Index (BI)

The level of independence in activities of daily living and functional mobility was assessed using the BI. This instrument assesses 10 activities: feeding (0–10 points), bathing (0–5 points), grooming (0–5 points), dressing (0–10 points), bowels (0–10 points), bladder (0–10 points), toilet use (0–10 points), transfers (bed to chair and back) (0–15 points), mobility (on level surfaces) (0–15 points), and stairs (0–10 points). Higher scores indicate greater functional independence, whereas lower scores reflect a greater inability to perform these activities. Scores range from 0 to 100 and are classified as follows: 0 to 20, total dependence; 21 to 60, severe dependence; 61 to 90, moderate dependence; 91 to 99, mild dependence; and 100, independence. The scale has demonstrated good reliability and validity [34].

##### Stroke Specific Quality of Life Scale (SS-QOL)

The quality of life of patients following stroke was evaluated using a condensed scale encompassing 12 domains: mobility, energy, upper limb functionality, work and productivity, mood, self-care, social roles, family roles, vision, language, cognition, and personality. Items are scored on a 5-point Likert-type scale (1 to 5). In the self-care domain, items are scored according to the level of help needed, from total help (1) to no help needed (5). In the language, mobility, upper limb functionality, vision, and work/productivity domains, items are scored according to the degree of trouble experienced, from a lot of trouble (1) to no trouble at all (5). In the energy, family roles, mood, personality, social roles, and cognition domains, items are phrased as statements and scored according to the level of agreement, from strongly agree (1) to strongly disagree (5). In all domains, higher scores indicate better functioning. Total scores range from 49 to 245, with higher scores indicating better quality of life. The scale has demonstrated good reliability and validity [35]. However, inter- and intra-rater reliability of the English-language version used has not been established in the literature.

### 2.5. Statistical Analysis

An a priori power analysis was conducted to calculate the required sample size based on an effect size of 0.25 (Cohen’s f, medium effect), a significance level of α = 0.05 (probability of committing a type I error), and statistical power of 95%. The sample size calculation was performed using G*Power version 3.1.9.7 (F tests: ANOVA, repeated measures, within factors), assuming a correlation of 0.5 among repeated measures, the default and most commonly used value in the absence of prior data. This yielded a minimum required sample of 43 patients. This estimate was increased by 20% to account for potential dropouts during the eligibility process, resulting in a total of 51 patients. Descriptive statistics were used to characterize the sample at baseline: sociodemographic and anthropometric variables (e.g., age, sex, BMI) were summarized using frequencies and percentages for categorical variables and means with standard deviations for continuous variables.

The R statistical package V 4.0.3 (R Core Team; Vienna, Austria) was used to analyze the experimental data. Three evaluation points were established: baseline assessment (T0), after the 12-week treatment (T1), and at the 3-month follow-up (T2). Prior to statistical analysis, the Shapiro–Wilk test was used to assess data normality, and Levene’s test was used to assess homogeneity of variances, for continuous variables only. Continuous response variables (BI, FMA, and SS-QOL) were analyzed using a generalized linear mixed-effects model for repeated-measures under the parametric assumption, equivalent to a repeated-measures ANOVA with patients as a within subject factor and the Friedman test under the nonparametric assumption. Categorical response variables (MAS and MRC) were analyzed using an ordinal logistic mixed-effects model. Treatment was included as the fixed-effects factor, and patient as the random-effects factor. The Bonferroni post-hoc test was used for pairwise comparisons. Differences were considered statistically significant at *p* ≤ 0.05 with a 95% confidence interval (CI). Additionally, partial eta squared (η^2^) was calculated to determine the effect size of the rehabilitation program, with effects classified as small (0.01), medium (0.06), or large (0.14) [36]. Results for the response variables are expressed as mean ± standard error for continuous variables and as median and interquartile range for categorical response variables.

## 3. Results

The baseline characteristics of the sample are shown in Table 1. In total, 51 post-stroke patients were included in the analysis (33 women, 18 men; mean age, 59.3 ± 18.7 years; range, 22–91 years). Most patients had an ischemic stroke (*n* = 46, 90.2%), and lesions were distributed almost equally between the left (n = 26, 51%) and right (n = 25, 49%) cerebral hemispheres. The mean time from symptom onset to baseline assessment was 27.5 ± 12.4 days (range, 12–63 days). All patients completed the treatment schedule. No complications associated with the treatment were observed, and no dropouts or withdrawals occurred.

**Figure 2:**
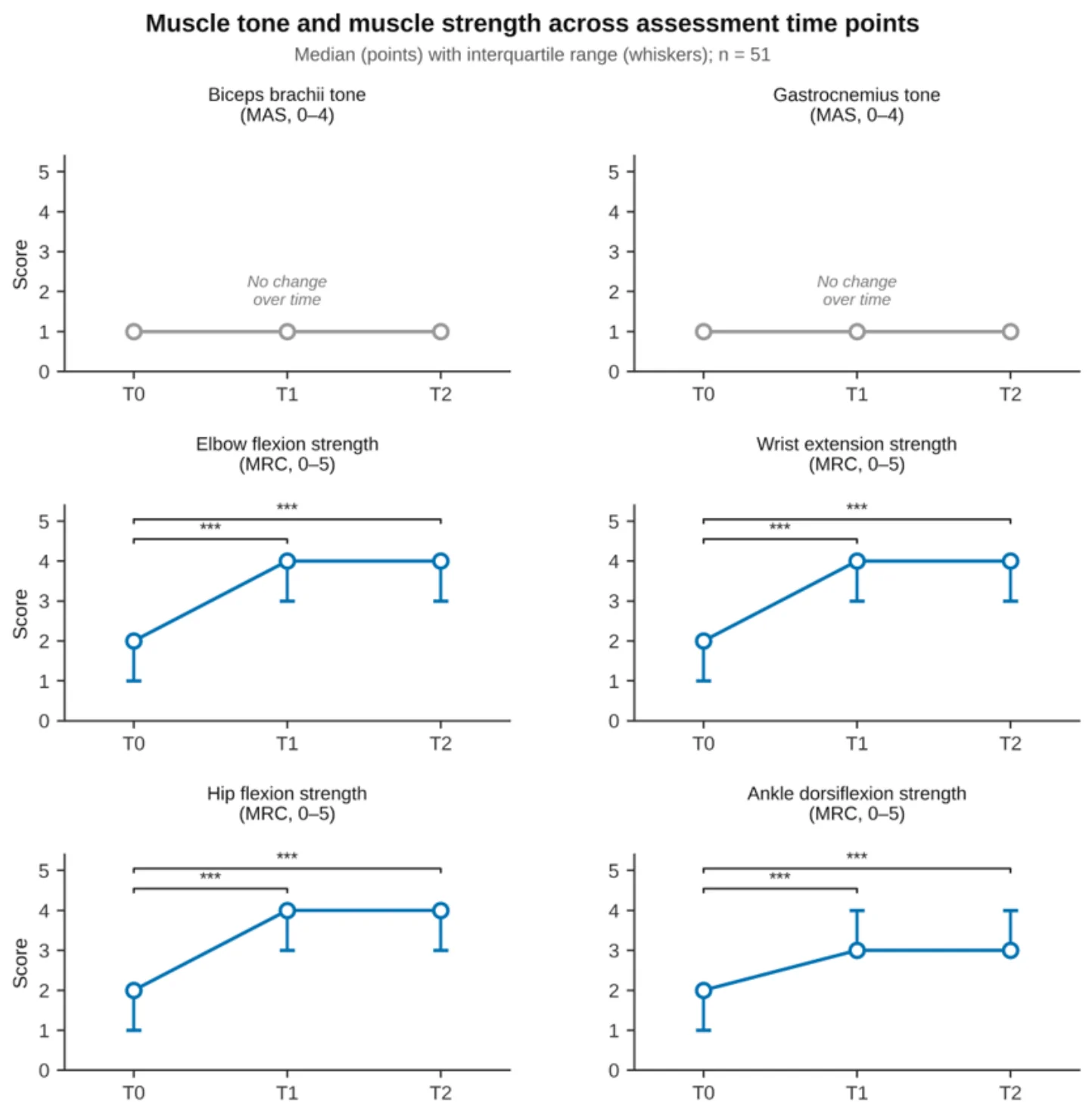
T0: baseline assessment; T1: after 12-week treatment; T2: 3-month follow-up. MAS, Modified Ashworth Scale; MRC, Medical Research Council Scale. Median (IQR) for MAS and MRC across T0, T1, and T2. *** *p* < 0.001 Significance was determined using a mixed-effects ordinal logistic regression model; pairwise comparisons were Bonferroni-adjusted.

Changes in clinical variables are shown in Table 2. Muscle tone, assessed using the MAS, showed no statistically significant changes at any of the three assessment time points for any of the muscles analyzed. The tone of the biceps brachii and gastrocnemius muscles remained unchanged at 1 (1–1) from T0 to T2 (*p* > 0.05), by contrast, significant improvements in muscle strength were observed from the initial assessment (T0) through 12 weeks after the intervention (T1) and up to the 3-month follow-up (T2) in all four muscle groups evaluated. (Figure 2) Elbow flexor strength increased from 2 (1–2) to 4 (3–4) at both T1 and T2 (*p* < 0.0001 for both comparisons). Wrist extensor strength increased from 2 (1–2) to 4 (3–4) at both T1 and T2 (*p* < 0.0001 for both comparisons). Hip flexor strength increased from 2 (1–2) to 4 (3–4) at both T1 and T2 (*p* < 0.0001 for both comparisons). Dorsiflexor strength increased from 2 (1–2) to 3 (3–4) at both T1 and T2 (*p* < 0.0001 for both comparisons). When T1 and T2 were compared, no significant differences were found in any muscle group, indicating that the strength gains achieved at 12 weeks were maintained at the three-month follow-up.

**Figure 3:**
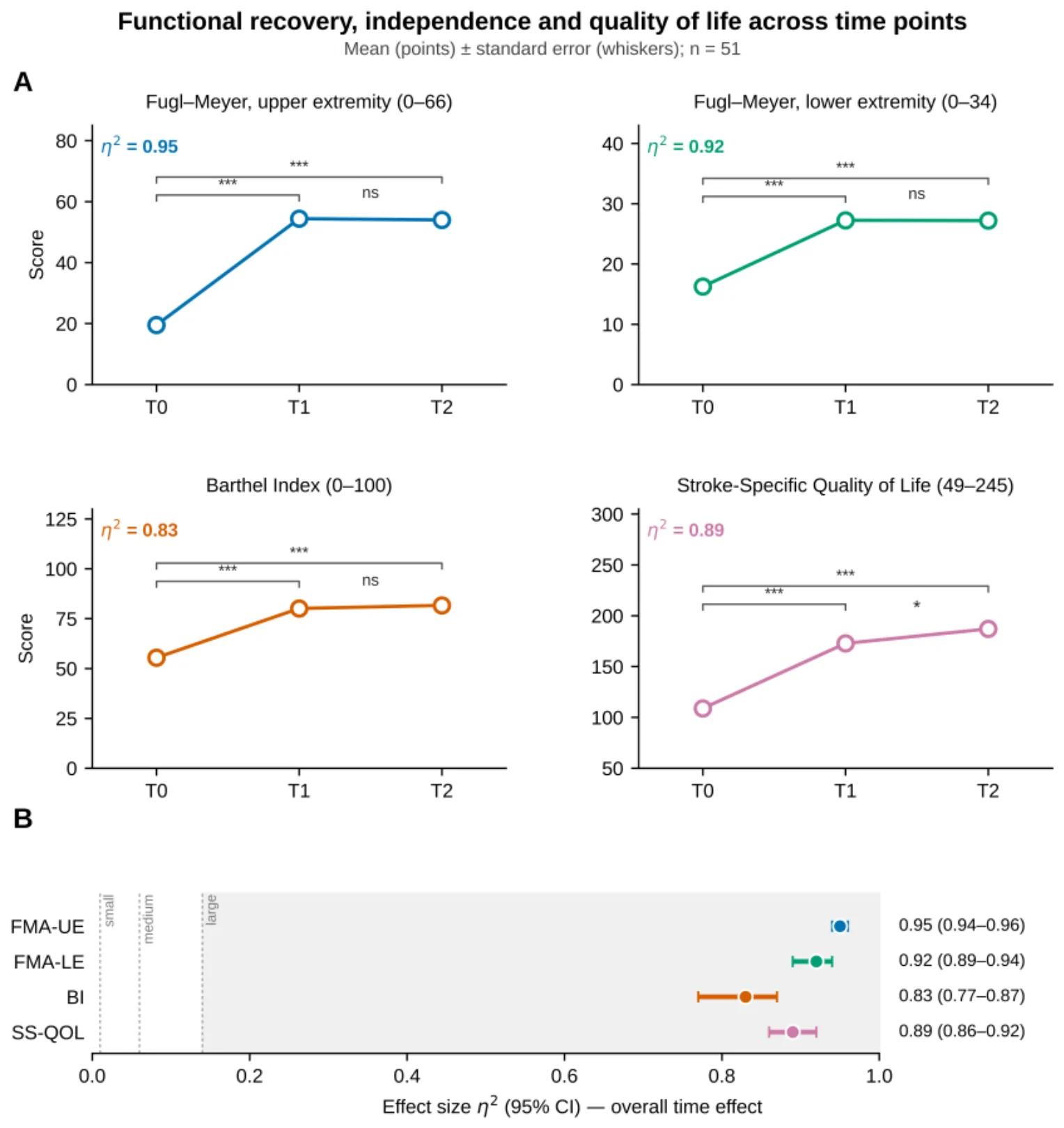
(**A**) Mean ± SE for (FMA-UE, FMA-LE, BI and SS-QOL across T0 (baseline assessment), T1 (after 12-week treatment), and T2 (3-month follow-up). Significance was determined using a generalized linear mixed-effects model for repeated measures, equivalent to a repeated-measures ANOVA with patient as the within subject factor; pairwise comparisons were Bonferroni-adjusted. (**B**) Effect size η^2^. *: *p* < 0.05, ***: *p* < 0.001, ns: not significant.

Table 3 shows changes in functional outcomes from baseline assessment (T0) to after the 12-week intervention (T1) and the 3-month follow-up (T2). At the 12-week follow-up, the FMA for the upper extremity (FMA-UE) showed an increase of 34.88 points (95% CI: 31.85–37.91; *p* < 0.0001), the FMA for the lower extremity (FMA-LE) showed an increase of 11.00 points (95% CI: 9.56–12.44; *p* < 0.0001), the BI showed an increase of 24.71 points (95% CI: 19.40–30.05; *p* < 0.0001), and the SS-QOL showed an increase of 63.90 points (95% CI: 51.60–76.30; *p* < 0.0001). At the 3-month follow-up, the FMA-UE showed an increase of 34.47 points (95% CI: 31.44–37.50; *p* < 0.0001; η^2^ = 0.95; 95% CI: 0.94–0.96), the FMA-LE showed an increase of 10.94 points (95% CI: 9.50–12.38; *p* < 0.0001; η^2^ = 0.92; 95% CI: 0.89–0.94), the BI showed an increase of 26.25 points (95% CI: 20.90–31.60; *p* < 0.0001; η^2^ = 0.83; 95% CI: 0.77–0.87), and the SS-QOL showed an increase of 78.30 points (95% CI: 65.90–90.60; *p* < 0.0001; η^2^ = 0.89; 95% CI: 0.86–0.92). At the 3-month follow-up, η^2^ values indicated a large effect size (η^2^ > 0.14) for all functional outcomes assessed (Figure 3).

## 4. Discussion

The 12-week rehabilitation program integrating thermal stimulation in addition to conventional therapy was associated with statistically significant improvements in voluntary muscle strength, motor function, functional independence, and quality of life in patients during the acute and subacute phases of first-ever stroke, while muscle tone remained stable throughout the intervention period, with no statistically significant changes. These gains were sustained at the 3-month follow-up without significant decline, with the sole exception of a reduction in ankle dorsiflexion strength between T1 and T2, which may be interpreted within the clinical context of unsupported ambulation during the follow-up period. Although post-stroke rehabilitation has been extensively studied, the existing literature has largely focused on isolated functional outcomes [37]. This study approached recovery from a perspective consistent with the International Classification of Functioning, Disability and Health (ICF) domains by simultaneously assessing motor function, functional independence, and quality of life, dimensions corresponding to body functions, activity, and participation, respectively, that previous research on thermal stimulation had not evaluated collectively [12,38]. In this context, to the authors’ knowledge, this is the first study to document simultaneous and significant improvements across all three domains, thereby expanding the available evidence to include the activity and participation dimensions that are central to optimal and meaningful recovery in post-stroke patients [38]. This is the first study to evaluate this protocol in Latin America [12].

The sociodemographic and clinical profile of the sample broadly aligns with available epidemiological data, including the predominance of women and the higher frequency of ischemic than hemorrhagic stroke [39]. Prior research applying thermal stimulation to the paretic upper limb in patients with acute stroke demonstrated significant improvements in sensory and motor outcomes compared with conventional therapy alone [40]. A study applying thermal stimulation to the lower limb in patients with acute stroke demonstrated superior outcomes in lower-limb motor function, balance, and ambulation when combined with standard rehabilitation protocols [41]. Longitudinal data from a 1-year follow-up study confirmed that the functional benefits of lower-limb thermal stimulation were maintained at 3 months, although most between-group differences attenuated by 6 and 12 months, with FMA-LE scores showing the most enduring effects [42]. The first systematic review and meta-analysis of this intervention confirmed statistically significant short-term gains in motor function, gait, and functional recovery when thermal stimulation was added to conventional therapy; however, its effect on functional independence, as measured by the BI, did not reach statistical significance, and health-related quality of life was not assessed in any included study [12]. A randomized controlled trial with 6-month follow-up found no significant advantage of noxious over innocuous thermal stimulation when both were combined with conventional rehabilitation, suggesting that the therapeutic effect may depend less on the temperature modality per se and more on the interaction between sensory input and simultaneous active motor practice [16]. No previous study of thermal stimulation in addition to conventional therapy has included the three domains covered by the ICF, which integrate functionality, independence, and quality of life.

Patients initiated the intervention at a mean of 27.5 days post-stroke, placing enrollment within the early subacute phase, a period characterized by the convergence of spontaneous biological recovery and heightened responsiveness to rehabilitation-induced neuroplasticity [43]. This therapeutic window is widely regarded as critical for maximizing functional recovery because it offers the greatest potential for reorganization of cortical and subcortical networks; evidence from experimental models and human neuroimaging studies indicates that early initiation of targeted rehabilitation during this period optimizes experience-dependent plasticity mechanisms, thereby increasing the probability of achieving clinically significant functional gains [43,44]. Within this neuroplastic window, spontaneous biological recovery and the effects of conventional therapy and thermal stimulation are expected to coincide and interact, such that the functional gains observed across the motor functional independence, and quality of life domains reflect the combined influence of these concurrent processes rather than an isolated effect of any single component.

Muscle strength across all four evaluated muscle groups showed large and statistically significant gains between T0 and T1 that were maintained at T2 in three of the four groups. The reduction in ankle dorsiflexion strength between T1 and T2, although not statistically significant, was of limited clinical magnitude and may plausibly reflect the greater neuromuscular demand placed on this distal muscle group during unsupported daily ambulation in the absence of structured therapeutic support.

Muscle tone remained stable throughout the study period, with no statistically significant changes at any evaluation point, a finding that should not be interpreted as an absence of therapeutic effect. This interpretation draws on a priori neurophysiological literature rather than on direct measurement in the present study. Voluntary motor recovery and muscle tone regulation are governed by distinct neural mechanisms, the former driven primarily by adaptive cortical reorganization along corticospinal pathways and the latter by reticulospinal hyperexcitability arising from maladaptive subcortical plasticity [45]. In the acute and subacute phases, the natural progression of stroke commonly involves a transition from initial flaccidity toward increasing passive resistance; therefore, the absence of increased tone in the present sample suggests that the rehabilitation program selectively promoted adaptive cortical plasticity without triggering the maladaptive subcortical changes underlying spastic hypertonia. This interpretation should be considered alongside the possibility that the stability of MAS scores partly reflects a floor effect and the limited sensitivity of this instrument in the present sample. This proposed dissociation is consistent with prior randomized evidence showing that thermal stimulation improves motor outcomes without significant between group differences in muscle tone [7,8,40]. This section consider possible mechanisms through which thermal stimulation in addition to conventional therapy may interact with the recovery process at each level.

Regarding motor function, the changes in FMA scores across the upper and lower extremity domains are consistent with the hypothesis that applying thermal stimulation immediately before active motor practice creates a favorable cortical environment for sensorimotor learning. Somatosensory input from peripheral thermal receptors ascends through the spinothalamic tract and dorsal columns and modulates the excitability of the primary motor cortex and associated premotor regions, lowering the activation threshold of the corticomotor networks engaged during voluntary movement [46]. In patients with stroke specifically, thermal stimulation increases corticomotor excitability, as measured by motor-evoked potential amplitude and cortical motor map size; these effects extend beyond the stimulation period itself and may therefore persist into the subsequent active training session [13]. Neuroimaging studies confirm that this stimulation recruits not only the primary somatosensory cortex but also the supplementary motor area, anterior cingulate cortex, and thalamus, structures directly implicated in motor planning and execution as well as sensorimotor integration [15]. When this state of cortical priming coincides with repetitive, task-oriented motor practice, the conditions for experience-dependent plasticity are substantially enhanced; the convergence of augmented sensory input and voluntary motor output during each session likely facilitated greater synaptic consolidation within motor networks than either modality could achieve independently [10,12,43]. The progressive structure of the conventional therapy component, which advanced systematically from proximal stabilization and active-assisted contractions in early sessions to resistance training, gait facilitation, and complex functional task execution, likely reinforced the cortical motor representations being reorganized through thermal priming at each stage [21].

The improvement in the BI during the intervention phase, representing a transition from moderate dependence toward independence in activities of daily living, reflects the extent to which the sensorimotor facilitation achieved through the combined protocol extended beyond isolated movement acquisition to support the relearning of goal-directed functional behaviors. Peripheral sensory input preceding motor practice facilitates activation of the sensorimotor circuits involved in functional task relearning, which may account for improvements in daily living performance beyond those attributable to motor recovery alone [46,47]. The repetitive and progressively complex nature of the functional tasks embedded in the conventional therapy component, encompassing transfers, weight-bearing, ambulation, and upper-limb daily activities, generated the use-dependent corticospinal modulation and changes in interhemispheric connectivity that underlie the consolidation of functional motor skills into meaningful daily performance [48]. The absence of statistically significant further change in the BI between T1 and T2 (*p* = 0.7720), occurring without a reclassification of dependence category, most plausibly reflects stabilization of the functional gains achieved during the intervention phase, consistent with the progressive integration of rehabilitation acquired skills into the patient’s unsupported daily routine, rather than continued accrual of new functional capabilities. This consolidation process, whereby performance of trained tasks becomes progressively automated through repeated execution in naturalistic contexts, is a recognized component of post-stroke functional recovery [48,49].

The continued, statistically significant improvement in SS-QOL scores between T1 and T2 (*p* = 0.0182) reflects a quality of life recovery trajectory that extended beyond the active intervention period and is both clinically and neurobiologically coherent. Quality of life after stroke is not a direct reflection of motor capacity; rather, it is a multidimensional construct shaped by cognitive function, emotional regulation, and social participation, each of which recovers along a distinct timeline. Impairments in attention, processing speed, and executive function represent the most proximate cognitive determinants of long-term well-being in stroke survivors [50,51]. Post-stroke depression, which is linked to disrupted prefrontal-limbic connectivity in proportion to depressive symptom severity, further compromises quality of life, indicating that recovery requires functional improvement alongside the gradual restoration of emotional regulation circuits [52]. Sustained participation in the rehabilitation program likely reinforced self-efficacy and intrinsic motivation through gains in motor control and functional independence, which are recognized as critical mediators of psychosocial adaptation after stroke [53]. This is consistent with evidence that disability severity and functional dependence are among the strongest longitudinal predictors of quality-of-life decline and that interventions reducing both factors can generate gains that accumulate beyond the end of active treatment as patients resume valued activities and social roles [54,55].

These findings have direct clinical implications for post-stroke neurorehabilitation. Combining strategies that increase peripheral afferent input with task-oriented motor training facilitates modulation of corticospinal pathways and enhances the neural circuits governing voluntary movement, contributing to improvements in strength and motor control of the affected hemibody [47,56]. The neuroplastic adaptations underlying these gains extend beyond the motor domain, as rehabilitation programs incorporating sensory stimulation have been associated with improvements in functional independence and quality of life, together constituting a more comprehensive recovery profile than motor training alone [57,58]. A clinically distinctive feature of the present protocol was the combination of early initiation and high treatment frequency, with five sessions per week delivered within the critical window of heightened neuroplasticity. This dosing strategy, which has not previously been documented in the thermal stimulation literature, may have maximized the cumulative sensory priming effect preceding each motor training session [44]. The concurrent improvements across all three ICF domains suggest a scope of effect not previously captured within a single study of thermal stimulation [37].

### Limitations of the Study

This study has several limitations that should be considered when interpreting the results. First, the case series design without a control group excludes statistical or empirical differentiation of relative contributions of spontaneous recovery, conventional therapy and thermal stimulation to the functional changes observed, and therefore prevents the establishment of a causal relationship between the intervention and these changes. Relatedly, the medium effect size (Cohen’s f = 25) used in a-prior sample size calculation was not derived from prior pilot data or an established minimal clinically important difference, as this would typically require comparative data from a randomized controlled trial: it was instead adopted as a conventional default commonly used in sample size planning when such data are unavailable [59]. Second, recruitment of patients from a single rehabilitation center limits the representativeness of the sample and restricts the generalizability of the findings to other clinical settings. Third, the exclusion of patients with cognitive impairment (Montreal Cognitive Assessment score < 20) or communication impairments such as aphasia, necessary to ensure valid self-reported outcomes, limits the generalizability of the findings to this common population of post-stroke patients in real world clinical practice. Fourth, comorbidity data were based on patients’ self-report rather than verified medical records, which may have led to underreporting, particularly for conditions requiring laboratory confirmation such as dyslipidemia. Fifth, no formally validated Spanish-language version of the SSQOL exists for use in Ecuador; items were translated into Spanish extemporaneously by the physiotherapist during the interview, following the structure of the original English-language version [35]. This may have introduced variability in item interpretation relative to a formally validated version. No study-specific inter-rater reliability coefficient was calculated and inter and intra rater reliability of the SSQOL has not been established in the literature. Sixth, the analysis was not stratified by stroke type (ischemic or hemorrhagic), preventing us from determining whether the response to the intervention differed between these two etiological subgroups. Seventh, the reported partial values of η^2^ reflect the overall effect of time in this single group, uncontrolled design, which may include contributions from spontaneous recovery and should not be interpreted as an effect isolated from treatment. Eighth, long-term clinical effects were not assessed; therefore, no conclusions can be drawn regarding the longer-term sustainability of the rehabilitation program’s effects. Activities undertaken by patients after treatment ended, such as home exercise, other therapies, medication changes, or botulinum toxin injections, were not recorded during the 3-month follow-up; therefore, the continued improvement observed between T1 and T2 cannot be attributed solely to the intervention and may also reflect the passage of time or psychosocial adaptation.

## 5. Conclusions

The addition of thermal stimulation to conventional therapy resulted in statistically significant short-term changes produced in motor function, functional independence, and quality of life among patients with a first acute or subacute stroke, and these improvements were maintained at the 3-month follow-up. Muscle tone remained stable throughout the study period, a finding consistent with increased adaptive cortical plasticity without maladaptive subcortical changes. These findings support the incorporation of thermal stimulation as a complementary component of neurorehabilitation programs during the subacute phase of stroke.

## Figures and Tables

**Table 1:** Baseline characteristics of patients.

Characteristics	Patients (n = 51)
Sex, number (%)	
Women	33 (64.7)
Men	18 (35.3)
Age (years), mean (SD)	59.3 (18.7)
Start days TS + CT after stroke, mean (SD)	27.5 (12.4)
Affected cerebral hemisphere, number (%)	
Left	26 (51)
Right	25 (49)
Type of stroke, number (%)	
Ischemic	46 (90.2)
Hemorrhagic	5 (9.8)
Height (cm), mean (SD)	162.2 (10.2)
Weight (kg), mean (SD)	66.9 (10.2)
BMI (kg/m^2^), mean (SD)	25.4 (2.9)
BMI classification, number (%)	
Normal	23 (45.1)
Overweight	25 (49)
Obesity	3 (5.9)
Employed activity before stroke, number (%)	
Unemployed	11 (21.6)
Employed	28 (54.9)
Independent	12 (23.5)
Hypertension, number (%)	
Yes	15 (29.4)
No	36 (70.6)
Diabetes Mellitus type 2, number (%)	
Yes	7 (13.7)
No	44 (86.3)
Dyslipidemia, number (%)	
Yes	2 (3.9)
No	49 (96.1)
Atrial fibrillation, number (%)	
Yes	0 (0)
No	51 (100)
Coronary artery disease, number (%)	
Yes	10 (19.6)
No	41 (80.4)
Tobacco consumption, number (%)	
Yes	8 (15.7)
No	43 (84.3)
Depression, number (%)	
Yes	7 (13.7)
No	44 (86.3)

SD: standard deviation; BMI: body mass index; TS: thermal stimulation; CT: conventional therapy.

**Table 2:** Changes in results for the tone muscle and muscle strength across assessment time points (n = 51).

Outcome Measure	T0 Median (IQR)	T1 Median (IQR))	Valor *p*	T2 Median (IQR))	Valor *p*	T1 Median (IQR)	T2 Median (IQR)	Valor *p*
Tone Biceps brachii (MAS)	1 (1–1)	1 (1–1)	>0.05	1 (1–1)	>0.05	1 (1–1)	1 (1–1)	>0.05
Tone gastrocnemius (MAS)	1 (1–1)	1 (1–1)	>0.05	1 (1–1)	>0.05	1 (1–1)	1 (1–1)	>0.05
Elbow flexion strength (MRC)	2 (1–2)	4 (3–4)	<0.0001	4 (3–4)	<0.0001	4 (3–4)	4 (3–4)	1.0000
Wrist extension strength (MRC)	2 (1–2)	4 (3–4)	<0.0001	4 (3–4)	<0.0001	4 (3–4)	4 (3–4)	1.0000
Hip flexion strength (MRC)	2 (1–2)	4 (3–4)	<0.0001	4 (3–4)	<0.0001	4 (3–4)	4 (3–4)	1.0000
Dorsiflexion strength (MRC)	2 (1–2)	3 (3–4)	<0.0001	3 (3–4)	<0.0001	3 (3–4)	3 (3–4)	1.0000

*p*-values shown are Bonferroni-adjusted for the three pairwise comparison within each outcome measure (T0-T1, T1-T2, T0-T2). Abbreviations: MAS: Modified Ashworth Scale; MRC: Medical Research Council; IQR: Interquartile range.

**Table 3:** Changes in results for the Fugl-Meyer Assessment (FMA), Barthel Index (BI), and Stroke Scale Quality of Life (SS-QOL) across assessment time points (n = 51).

Outcomes Measure	T0 Mean (SE)	T1 Mean (SE)	Mean Difference (CI 95%)	*p *Value T0-T1	T2 Mean (SE)	Mean Difference (CI 95%)	*p *Value T0-T2	T1 Mean (SE)	T2 Mean (SE)	Mean Difference (CI 95%)	*p *Value T1-T2	ƞ^2^ (CI 95%)
FMA-UE (0–66)	19.51 ± 0.88	54.39 ± 0.89	34.88 (31.85–37.91)	<0.0001	53.98 ± 0.94	34.47 (31.44–37.50)	<0.0001	54.39 ± 0.89	53.98 ± 0.94	−0.41 (−3.44–2.62)	0.9446	0.95 (0.94–0.96)
FMA-LE (0–34)	16.25 ± 0.53	27.25 ± 0.36	11.00 (9.56–12.44)	<0.0001	27.196 ± 0.39	10.94 (9.50–12.38)	<0.0001	27.25 ± 0.36	27.196 ± 0.39	−0.06 (−1.50–1.38)	0.9949	0.92 (0.89–0.94)
BI	55.39 ± 1.71	80.10 ± 1.43	24.71 (19.40–30.05)	<0.0001	81.65 ± 1.64	26.25 (20.90–31.60)	<0.0001	80.10 ± 1.43	81.65 ± 1.64	1.55 (−3.80–6.89)	0.7720	0.83 (0.77–0.87)
SS-QOL	108.78 ± 3.49	172.73 ± 3.60	63.90 (51.60–76.30)	<0.0001	187.04 ± 3.93	78.30 (65.90–90.60)	<0.0001	172.73 ± 3.60	187.04 ± 3.93	14.30 (2.00–26.60)	0.0182	0.89 (0.86–0.92)

*p*-values shown are Bonferroni-adjusted for the three pairwise comparison within each outcome measure (T0-T1, T1-T2, T0-T2). Abbreviations: FMA-UE: Fugl-Meyer Assessment upper extremity; FMA-LE: Fugl-Meyer Assessment lower extremity; T0 = baseline assessment; T1 = after 12-week treatment; T2 three months of follow-up; SE: Standard error; CI 95%: confidence intervals 95%; η^2^: effect size.

## Data Availability

The data presented in this study are available upon reasonable request to the corresponding author. The data are not publicly available due to ethical and privacy restrictions, as the participants’ informed consent limits their use to the proposed research and to subsequent research along the same lines, subject to approval by a Human Research Ethics Committee endorsed by the Ministry of Public Health of Ecuador. The study was approved by the Ethics Committee of the University of the Americas (2024-OBS-008).

## Supplementary Materials

The following supporting information can be downloaded at https://www.mdpi.com/article/10.3390/healthcare14213673/s1: Table S1: Detailed Intervention Protocol.
