# Supplementary material for: Thermal Stimulation in Addition to Conventional Therapy on Functional Recovery After Stroke: A Case Series
Source: Healthcare (Basel). 2026 Oct 29;14(21):3673. doi: 10.3390/healthcare14213673 (PMC13763272; doi:10.3390/healthcare14213673)
Supplement: Supplementary file 1 [file healthcare-14-03673-s001.zip › healthcare-4619357-supplementary.pdf]

Supplementary Table S1. Detailed Intervention Protocol

Session	Contents	Position
**Sessions 1–2**	**Phase 1 – Thermal Stimulation**<br>● Hot compress (>45 °C) applied for 15 seconds.<br>● Cold compress (<15 °C) applied for 30 seconds.<br>● 30-second rest interval between applications.<br>● 10 repetitions per cycle.<br>● 60-second rest between cycles.<br>**Phase 2 – Physical Therapy**<br>● Facilitation of key control points of the pelvis and scapular girdle.<br>● Execution of directed and guided movements with visual and tactile feedback.<br>● Active-assisted muscle contractions of flexion, extension, abduction, adduction, and rotations of the affected hemibody.<br>● Righting reaction training with cephalic-to-caudal movement sequences.<br>● Active-assisted trunk rotations to promote pelvic and scapular dissociation.	**Thermal Stimulation** Thermal stimulation was applied locally to the palm of the hand and the sole of the foot on the hemiplegic side.<br>Two cycles applied.<br>**Physical Therapy** Supine and right and left lateral decubitus positions.<br>Moderate intensity, low number of repetitions (8–10), with frequent rest intervals to monitor fatigue and cardiovascular response to exercise.
**Sessions 3–4**	**Phase 1 – Thermal Stimulation**<br>● Hot compress (>45 °C) applied for 15 seconds.<br>● Cold compress (<15 °C) applied for 30 seconds.<br>● 30-second rest interval between applications.<br>● 10 repetitions per cycle.<br>● 60-second rest between cycles.<br>**Phase 2 – Physical Therapy**<br>● Postural symmetry through postural adjustments and awareness with visual feedback.<br>● Reaching for objects at midline level with auditory and visual stimuli.	**Thermal Stimulation** Thermal stimulation was applied locally to the palm of the hand and the sole of the foot on the hemiplegic side.<br>Two cycles applied.<br>**Physical Therapy** Sitting, quadruped, half-kneeling, and static standing positions.

Session	Contents	Position
	• Balance reaction training with weight displacement in quadruped and sitting positions.<br>• Alternating activation of upper and lower limbs in a four-point kneeling position to engage crossed muscle chains.<br>• Balance reaction training in a half-kneeling position with activation of the abdominal girdle, gluteal muscles, and progressive weight bearing.<br>• Active-assisted muscle contractions of the upper and lower limb musculature for functional and activities-of-daily-living tasks.<br>• Introduction of bilateral weight bearing in standing.	Moderate intensity, increased number of repetitions (12–15), moderate manual resistance, and introduction of dual-task exercises (attention + movement).
**Sessions 5–12**	**Phase 1 – Thermal Stimulation**<br>• Hot compress (>45 °C) applied for 15 seconds.<br>• Cold compress (<15 °C) applied for 30 seconds.<br>• 30-second rest interval between applications.<br>• 10 repetitions per cycle.<br>• 60-second rest between cycles.<br>**Phase 2 – Physical Therapy**<br>• Facilitation of gait pattern with correction of compensatory strategies and use of support points.<br>• Progressive resistance exercises in global movement patterns (diagonals) of the lower limb during single-leg support.<br>• Muscle chain strengthening exercises, balance training, and gait re-education.<br>• Transfer training (supine to sitting, sitting to standing).	**Thermal Stimulation** Thermal stimulation was applied locally to the palm of the hand and the sole of the foot on the hemiplegic side.<br>Two cycles applied.<br>**Physical Therapy** Sitting, standing, and gait positions.<br>Moderate intensity with a higher number of repetitions (15–20, exceeding 4–5 sets), introduction of light to moderate external resistance, and progression toward tasks

Session	Contents	Position
	• Balance, righting, and protective reaction training with lateral and anteroposterior weight shifts.<br>• Progressive assisted gait training (from orthotic aids to supervised independent ambulation).<br>• Execution of progressive functional tasks (walking while carrying objects, ascending low steps, navigating ramps).	of greater functional complexity.
